# Heart Rate Variability and Complementary Medicine

**DOI:** 10.1155/2014/395485

**Published:** 2014-02-13

**Authors:** Gerhard Litscher, Wei He, Seung-Ho Yi, Lu Wang

**Affiliations:** ^1^Stronach Research Unit for Complementary and Integrative Laser Medicine, Research Unit of Biomedical Engineering in Anesthesia and Intensive Care Medicine, TCM Research Center Graz, Medical University of Graz, 8036 Graz, Austria; ^2^Institute of Acupuncture and Moxibustion, China Academy of Chinese Medical Sciences, Beijing 100700, China; ^3^Kyung Hee University, Seoul 130-701, Republic of Korea

Heart rate variability (HRV) is a parameter of the neurocontrol of the heart and is used more and more in recent scientific research and practice, not only in Western medicine, but also in evidence-based traditional medicine. Today innovative research including the latest recording technology and also artificial intelligence techniques are used for data acquisition and data analysis of HRV in acupuncture and herbal medicine research.

The scope of HRV is not yet completely clear, but it is known that there are intraindividual and interindividual variances and that heart rate variation depends on age. It becomes less random with the aging process and the appearance of age-related diseases. Apart from age, circadian variations (sleep-wake cycle), physical condition, and mental and physical exertion are important influencing factors. HRV can also be affected by diverse conditions such as age-related diseases and so-called lifestyle diseases like diabetic neuropathy, renal failure, essential hypertension, cardiac disorders, coronary artery disease, or intracranial lesions. In all cases, different medications have to be taken into account.

HRV can be used as a globally reliable indicator of the state of health. However, it could be demonstrated in this special issue that in special syndromes like stress or burnout one can counteract this process using different preventive methods like acupuncture.

This special issue focuses on the latest innovative aspects concerning HRV and complementary medicine. Altogether, 25 manuscripts underwent a review process. Sixteen articles were accepted for publication. Some of the papers report results of HRV in combination with different kinds of acupuncture (manual needle acupuncture, laser acupuncture, and electroacupuncture).

Important topics of this special issue are basic and clinical HRV research in complementary medicine, the development of innovative HRV-related concepts for assessing the state of health, new methods for the quantification of HRV, HRV and practical implications in traditional medicine, and telemedicine and HRV. This special issue contains basic research studies and clinical studies concerning menstrual pain and menstrual distress, burnout syndrome, depression, and insomnia, animal experimental studies in rats and dogs, and a review article.

